# Rectal Invasion by Metastatic Prostate Adenocarcinoma

**DOI:** 10.7759/cureus.15569

**Published:** 2021-06-10

**Authors:** Anshu Wadehra, Samer Alkassis, Aliza Rizwan, Omid Yazdanpanah

**Affiliations:** 1 Internal Medicine, Wayne State University/Detroit Medical Center, Detroit, USA

**Keywords:** prostate cancer, rectal mass, rectal metastasis, rectal pain, sigmoidoscopy

## Abstract

In men, prostate cancer is the second most diagnosed cancer worldwide. Typical sites for metastasis include bone, lung, and liver. Prostate cancer with gastrointestinal involvement, particularly rectal, has been rarely reported in the literature. As patients with prostate cancer with rectal invasion may present with symptoms similar to those of other gastrointestinal pathologies, such as anal fissures and rectal carcinoma itself, misdiagnosis and delays in care can result. Direct visualization of the rectum via endoscopy, along with biopsy, allows clinicians to make an accurate and timely diagnosis in patients with prostate cancer with rectal involvement, which in turn leads to broader available treatment options.

## Introduction

Prostate cancer is the second most common cancer in men worldwide [1]. Although localized disease is the most common finding at the time of diagnosis, some patients have evidence of metastatic prostate cancer at presentation. The most frequent location of metastasis is bone, particularly the axial skeleton, followed by lung and liver [2]. Gastrointestinal, especially rectal, involvement by prostate cancer has been rarely reported in the literature [3]. Invasion to the rectum occurs through three proposed routes; either direct invasion through the fascia, lymphatic spreading, or iatrogenic seeding of cancer cells during transrectal biopsy [4]. Predominant lower gastrointestinal symptoms, which indicate advanced prostate carcinoma, and the appearance of a rectal mass on colonoscopy may lead to misdiagnosis as a rectal carcinoma and resultant inappropriate treatment [5,6]. We report a case of an elderly gentleman who presented with worsening rectal pain and was found to have a rectal mass on sigmoidoscopy with biopsy showing prostate adenocarcinoma.

## Case presentation

The patient was a 90-year-old male with a history of hypertension, type 2 diabetes mellitus, and prostate adenocarcinoma under active surveillance, who presented with chronic constipation. His symptoms were initially controlled with stool softeners and enemas. On follow-up visit, the patient was noted to have one week of rectal pain which was exacerbated by defecation. He also endorsed decreased appetite, without any changes to his weight. He denied any abdominal pain, nausea, or vomiting. There was no abdominal tenderness on physical examination. However, there was significant tenderness noted during digital rectal examination, along with brown stool in the rectal vault.

**Figure 1:**
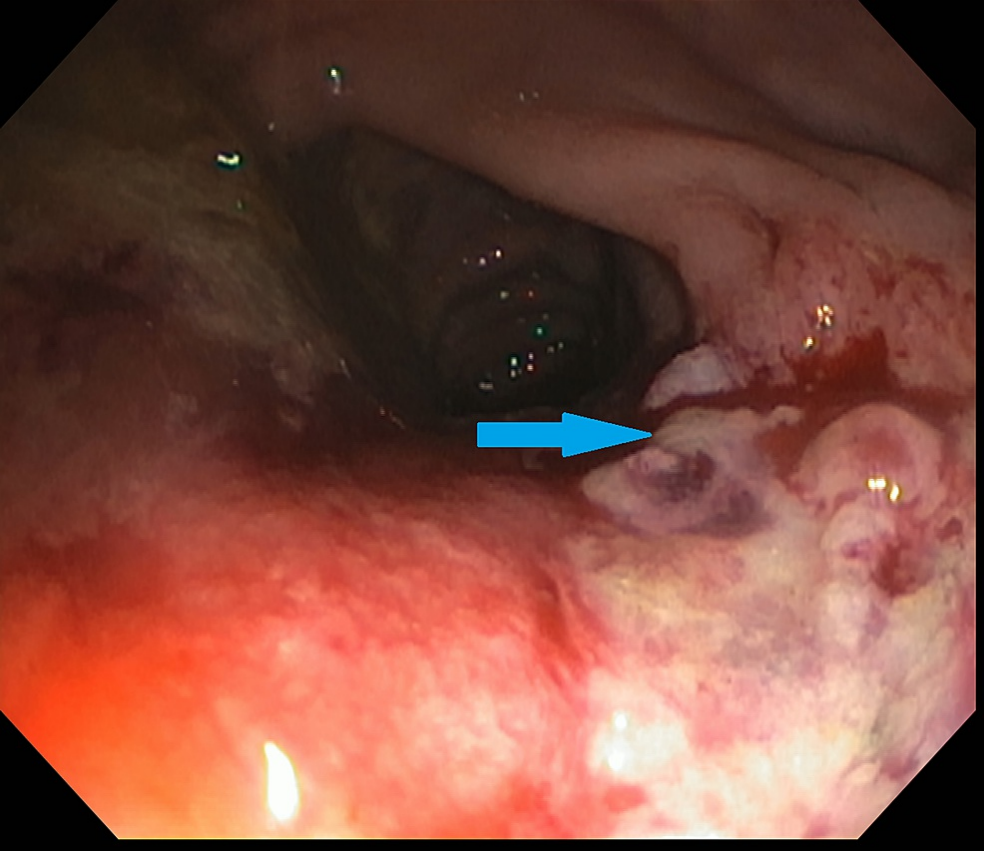
Flexible sigmoidoscopy significant for ulcerated rectal mass (blue arrow).

**Figure 2:**
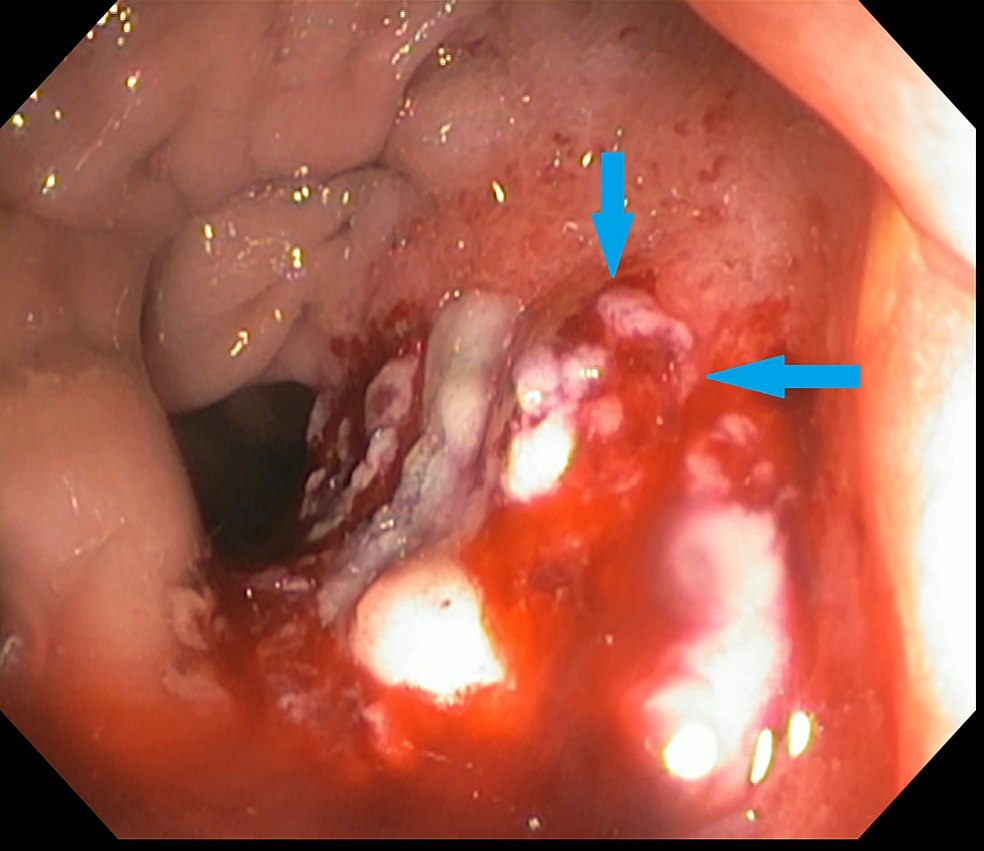
Flexible sigmoidoscopy significant for ulcerated rectal mass (blue arrows).

As a result, the patient was initially managed for a presumed anal fissure with a high-fiber diet, topical analgesics, and stool softeners. Despite this therapy, the patient's rectal pain did not improve. MRI of the abdomen and pelvis was obtained which was significant for moderate asymmetric thickening of the rectum anteriorly concerning for a possible neoplastic process. Despite the patient having a normal colonoscopy two years prior, a flexible sigmoidoscopy was performed. An ulcerated, friable rectal mass 8 cm from the anal verge was found (Figures 1-2).

The mass occupied less than 25% of the circumference of the colon. Biopsy was significant for moderately differentiated adenocarcinoma consistent with prostatic origin. Prostate-specific antigen (PSA) was less than 0.01 ng/mL. The patient was deemed to not be a candidate for radiation therapy. The patient additionally declined chemotherapy given his advanced age. He was further referred to palliative care for management of his symptoms.

## Discussion

Prostate cancer is one of the most commonly encountered malignancies in men worldwide, carrying an increased risk of cancer related morbidity and mortality, with an estimated 366,000 deaths annually [1]. In prostate cancer, the cells of the prostate gland mutate into cancer cells. Mutation is usually in the p53 gene or alteration of the Akt kinase signaling [7]. The presentation of prostate cancer varies from localized cancer to that involving distant metastases [8]. Most patients are asymptomatic at the time of diagnosis, however, lower urinary tract symptoms such as urinary urgency and nocturia, as well as hematuria can occur [9].

Prostate cancer commonly metastasizes to bone, liver, and lung [2]. It is rare for prostate cancer to metastasize to areas like the brain, breast, and gastrointestinal tract [3]. In spite of being in close vicinity, prostate cancer rarely invades the rectum due to the presence of a membranous barrier, Denonvilliers’ fascia, that separates the prostate gland from the rectum [4]. Despite this fact, there have been a handful of case reports highlighting rectal invasion by prostate cancer [3-5,10]. Rectal invasion by prostate cancer may occur via iatrogenic seeding during needle biopsy, direct invasion, or lymphatic metastasis into the rectum [4]. The presentation of rectal involvement may include obstructive symptoms, such as changes in stool caliber and constipation, as well as rectal bleeding [11]. As these symptoms may be present in a number of other conditions, including rectal carcinoma itself, patients may initially be mismanaged and have resultant delays in care [5,6].

Prostatic adenocarcinoma with rectal metastasis not only presents clinically similar to rectal adenocarcinoma but also shares morphological similarities with primary rectal cancer, which makes diagnosis even more challenging [12]. Macroscopically, prostate cancer with rectal metastasis may present as a circumferential mass usually 3-4 cm away from the anus [13]. Microscopically, however, it may present with a wide range of appearances, including small glands, diffuse individual cell infiltration, cribriform structures, and foamy glands with lipid accumulation [13]. PSA and carcinoembryonic antigen (CEA) levels can aid in further differentiating prostate cancer with rectal metastasis from primary rectal adenocarcinoma, although the PSA level may not always be found to be elevated with prostate adenocarcinoma, as was the case in our patient [13].

Various treatment options for prostate cancer exist such as external beam radiation therapy, hormone therapy, and surgery including radical prostatectomy, which may then be followed by radiation therapy and/or hormone therapy [14]. However, for those patients with rectal involvement, the prognosis is generally unfavorable because of advanced carcinoma [5]. As a result, in patients presenting with new-onset rectal pain and a known history of prostate cancer, it is imperative for clinicians to pursue direct visualization of the rectum via endoscopy in order to establish an accurate diagnosis, given the possibility of rectal wall invasion by prostate cancer. Early and accurate diagnosis of this condition will allow for overall broader treatment options as well as reducing delays in care.

## Conclusions

Prostate cancer with rectal involvement is a rare entity. Patients may present with symptoms similar to those of an anal fissure or rectal carcinoma itself, leading to initial misdiagnosis. Clinicians must be aware of the importance of accurate diagnosis in this group of patients to reduce delays in care and allow for broader treatment options. In addition, even in spite of a recent normal lower gastrointestinal endoscopy, clinicians should have a low threshold to pursue endoscopic evaluation of the rectum in prostate cancer patients presenting with new-onset rectal pain.
